# Association Between Current Physical Activity and Current Perceived Anxiety and Mood in the Initial Phase of COVID-19 Confinement

**DOI:** 10.3389/fpsyt.2020.00729

**Published:** 2020-07-23

**Authors:** Rubén López-Bueno, Joaquín Calatayud, Yasmin Ezzatvar, José A. Casajús, Lee Smith, Lars L. Andersen, Guillermo F. López-Sánchez

**Affiliations:** ^1^ Department of Physical Medicine and Nursing, University of Zaragoza, Zaragoza, Spain; ^2^ Department of Musculoskeletal Disorders, National Research Centre for the Working Environment, Copenhagen, Denmark; ^3^ Exercise Intervention for Health Research Group (EXINH-RG), Department of Physiotherapy, University of Valencia, Valencia, Spain; ^4^ Faculty of Health Sciences, University of Zaragoza, Zaragoza, Spain; ^5^ Cambridge Centre for Sport and Exercise Science, Anglia Ruskin University, Cambridge, United Kingdom; ^6^ Faculty of Sport Sciences, University of Murcia, Murcia, Spain

**Keywords:** physical activity, mental health, Spain, adults, COVID-19

## Abstract

The World Health Organization (WHO) has declared a world pandemic due to COVID-19, and several enacted measures such as compulsory confinement may have collateral consequences on both physical and mental health. We aimed to investigate associations between current physical activity (PA) and current perceived anxiety and mood among a sample of Spanish adults confined due to COVID-19 restrictions of movement. Using an online survey, we collected data on the Spanish adult population regarding health habits during the first days of enacted confinement. A total of 2250 participants (54.8% women) aged 35.3 (SD 13.6) completed the survey, which included questions about sociodemographic characteristics (i.e. age, gender, civil status, education, and occupation), health habits (i.e. prior PA, alcohol consumption, smoking, screen exposure, and sleep hours) and COVID-19 confinement context (i.e. number of isolation days, solitude, and exposure to COVID-19). Physical Activity Vital Sign (PAVS) short form was used to estimate weekly minutes of PA, whereas a single-item question was used to assess both current perceived anxiety and mood. We conducted weighted binomial logistic regressions to check associations between current adherence to WHO guidelines of PA and current perceived anxiety and mood of confined adults. Significant inverse associations between overall adherence to PA and current perceived anxiety in the final adjusted model (OR, 0.66; 95% CI, 0.54–0.79) as well as in several subgroup analyses such as younger women were observed. In addition, a borderline significant inverse association was found between current PA and current perceived worse mood when fully adjusted (OR, 0.82; 95% CI, 0.68–1.00); this association was significantly stronger in women than men. The results of the present study indicate that current PA adherence to WHO guidelines in the initial phase of COVID-19 confinement associates with both lower current perceived anxiety and lower current perceived worse mood among a sample of Spanish adults.

## Introduction

Coronavirus disease 2019 (COVID-19) is considered a global pandemic by the World Health Organization (WHO) since March 11, 2020 (1). The current number of cases (date 4 April 2020) globally diagnosed is 972,203, which has resulted in 50,321 people dead so far (2). Spain is one of the most inflicted countries, with a total of 124,736 cases diagnosed, resulting in 11,744 deaths until now (3).

Due to this situation, in an attempt to fight the spread of this virus, the Spanish Government approved a period of confinement as of 15^th^ March of 2020; some of the immediate consequences of the confinement period are that people have to stay at home more than usual, interrupt their usual activities and change their habits (4). Also, telework has been recommended to many Spanish people during this period and, in this context, to perform PA at home has been further recommended (4, 5).

As prolonged home stays can increase behaviors that lead to inactivity, maintaining regular PA, and routinely exercising in a safe home environment is an important strategy for healthy living during the coronavirus crisis (6). Doing regular PA during COVID-19 confinement is also very important for mental health because moderate PA improves mood (7) and helps to prevent anxiety and depression (8). Low PA is associated with feelings of loneliness and lack of social support (9, 10). In this specific situation of isolation created by the confinement, special attention should be paid to mental health, particularly in those living alone and/or experiencing loneliness (11); only during the initial phase of the COVID-19 outbreak in China, 53.8% of respondents rated the psychological impact as moderate-severe, with 16.5% reporting moderate-severe depressive symptoms and 28.8% reporting moderate-severe anxiety symptoms (12).

Furthermore, the use of PA to prevent mental health problems during the confinement is a very adequate strategy, because previous research has found that PA produces not only long-term benefits in mental health but also immediate psychological benefits for mood and anxiety due to the acute effects of PA (13–15). Due to these reasons, PA has now been recommended as a therapy to fight against the mental and physical consequences of COVID-19 confinement (16).

While people should perform at least the PA recommended by WHO (15, 16), also during the confinement period, this may be extremely difficult to achieve. The recommendation of WHO for adults is to do at least 150 minutes of moderate-intensity PA per week, or 75 minutes of vigorous-intensity PA per week, or an equivalent combination of moderate- and vigorous-intensity physical activity achieving at least 600 MET-minutes/week (17, 18). However, recent recommendations suggest that, during the confinement, people should do even more PA than these recommendations to compensate for the increase in sedentary time at home (16). While many people are unlikely to exceed this threshold of PA, the adverse influence on mental health needs to be investigated. Also, we found PA particularly critical to analyze since prior research has suggested the importance of PA during the COVID-19 confinement (6, 16).

In view of this context, it is necessary and urgent to analyze the association between PA and mental health in adults confined due to COVID-19, in order to prevent mental health problems associated with the expected reduction of PA during the confinement. Thereby, the main objective of this study was to evaluate the association between current PA and current perceived both anxiety and mood among Spanish adults confined due to COVID-19.

## Methods

We conducted a cross-sectional study to check associations regarding current PA and both current perceived anxiety and mood among a sample of the confined Spanish population due to COVID-19. Retrieved data from an online health survey carried out during the Spanish confinement period were used.

### The Survey

Data were collected through a web-form link during the period 22^nd^ to 29^th^ of March, 2020 (i.e. from the seventh official day of Government-enacted national confinement). The link was launched on social media encouraging Spanish resident users to answer the survey. According to server analytics, 2,850 media users covering all the Spanish regions were asked to participate. Participants were previously informed about the aims of the study, gave informed consent to participate, and confirmed that they were in an isolation situation due to COVID-19 enacted restrictions. The survey was anonymous and treated accordingly to Spanish legislation as regards general data protection. At the end of the survey, participants were provided with information on how to exercise at home and its potential benefits. The study was carried out accordingly with the principles of the World Medical Declaration of Helsinki, and got the approval of the Ethics Committee of Research in Humans of the University of Valencia, with register code 1278789. The reporting of this study adheres to the Strengthening the Reporting of Observational Studies in Epidemiology statement (STROBE) (19).

### Current Physical Activity

Estimations of current PA were conducted using the Physical Activity Vital Sign (PAVS) short version. The participants answered two questions concerning the number of days and minutes a week they did PA before and during a confinement week, with possible answers comprising 0, 1, 2, 3, 4, 5, 6, or 7 days of PA per week and 10, 20, 30, 40, 50, 60, 90, and 150 or more daily minutes (20, 21). To implement PAVS to that particular context of population confinement, the word “isolation” replaced the word “usual” when referring to the week they performed PA. Accordingly to PAVS original procedure, weekly minutes of PA were estimated by multiplying days with minutes.

Participants were categorized into those not achieving WHO recommended guidelines regarding moderate-intensity aerobic PA (i.e. those who did not perform a minimum of 150 weekly minutes of PA) and those who did.

### Current Perceived Anxiety and Mood

Outcome variables were estimated through the following questions: “how do you assess your anxiety level during the COVID-19 confinement?”, with possible responses comprising “lower than before the COVID-19 confinement”, “equal than before the COVID-19 confinement”, or “higher than before the COVID-19 confinement”. As regards mood, we asked the participants through the following question: “how do you assess your mood during the COVID-19 confinement?”, and possible answers including the following options: “worse than before the COVID-19 confinement”, “equal than before the COVID-19 confinement”, or “better than before the COVID-19 confinement”. We later categorized the current perceived anxiety variable into two groups; those having lower or equal levels of current perceived anxiety and those having higher levels. On the other hand, the current perceived mood variable were also categorized into those having a better or equal current perceived mood and those having worse.

### Covariates

Following previous studies (22–24), the present work also estimated age, gender, socioeconomic status (marital status, occupation, and education), and health habits (smoking, alcohol consumption, hours sleeping, and time exposed to screens). In addition, other variables concerning the confinement context were also controlled: number of days isolated because of COVID-19 confinement, whether participants were living alone during COVID-19 confinement, and, last, whether they were exposed or infected with COVID-19. Self-reported answers were categorized as follows: marital status (“married or having a partner” or “not married neither having a partner”), occupation (“employed” or “not employed”), education (“having a university degree” or “not having a university degree”), smoking habits (“current smoker” or “not a current smoker”), alcohol consumption (“usual”, “moderate” or “never”), solitude during the COVID-19 confinement (“alone while confined” or “not alone while confined”), and exposure to COVID 19 (“infected with COVID-19 or close to an infected person” or “not exposed”). Quantitative control variables were reported as follows: time sleeping (“number of average hours sleeping while COVID-19 confinement”), time exposed to screens (“number of average hours exposed to screens such as watching TV, cell phone, and tablet during COVID-19 confinement”), and number of days isolated because of the COVID-19 confinement (“number of days isolated since the 15^th^ of March, 2020”, i.e. the first enacted COVID-19 confinement day in Spain).

### Statistical Analyses

Statistical analyses were conducted using the Statistical Package for Social Sciences (SPSS) version 23.0 (SPSS Inc., Chicago, IL). The Kołmogorov–Smirnov test was applied to check normality. We computed weighted binomial logistic regression tests to check associations between the current adherence to WHO guidelines regarding PA and current perceived both anxiety and mood in the COVID-19 confinement period, providing odds ratios (ORs) and 95% confidence intervals (CIs) for the overall sample. Stratified analyses were also conducted to assess such associations concerning age (i.e. cutoff point of 45 years old, which is a critical point regarding mental conditions for both sexes in the Spanish population) (25), and gender. Participants with missing data in any of the variables were discarded for the study (n=60). Levels of significance were set at P < 0.05.

## Results

A total of 2250 participants aged 35.3 (SD 13.6) completed the survey. Descriptive statistics of the sample are illustrated in **Table 1**, in which 1232 participants (54.8%) are women, and 210 (9.3%) declared to be infected with COVID-19 or being close to someone who was. On average, participants were confined for 7.2 days (SD 2.9), and 168 (7.5%) were alone while confined. The mean for weekly minutes of PA while confined was 182.5 (SD 180.7).

**Table 1:** Characteristics of the study population.

N = 2,250	n (%)	Mean (SD)
Age (y)		35.3 (13.6)
**Gender**		
Men	1,018 (45.2)	
Women	1,232 (54.8)	
**Marital status**		
Married or having a partner	1,105 (49.1)	
Not married or having a partner	1,145 (50.9)	
**Occupation**		
Employed	1,399 (62.2)	
Not employed	851 (37.8)	
**Education**		
Holding a university degree	1,377 (61.2)	
Not holding a university degree	873 (38.8)	
**Alcohol consumption**		
Never	634 (28.2)	
Moderate	1,446 (64.2)	
Usual	170 (7.6)	
**Smoker**		
No	1,928 (85.7)	
Yes	322 (14.3)	
Sleep time (h)		7.9 (1.1)
Screen time (h)		5.8 (2.3)
**Exposure to COVID-19**		
Yes	210 (9.3)	
No	2,040 (90.7)	
**Alone while COVID-19 lockdown**		
Yes	168 (7.5)	
No	2,082 (92.5)	
Days isolated from COVID-19		7.2 (2.9)
PA weekly minutes while isolated from COVID-19		182.5 (180.7)
**WHO PA recommendations while isolated from COVID-19**		
Yes	1,142 (50.8)	
No	1,108 (49.2)	
PA weekly minutes before isolated from COVID-19		222.4 (190.9)
**WHO PA recommendations before isolated from COVID-19**		
Yes	1,374 (38.9)	
No	876 (61.1)	
**Current perceived higher anxiety**		
Yes	970 (43.1)	
No	1,280 (56.9)	
**Current perceived worse mood**		
Yes	984 (43.7)	
No	1,266 (56.3)	

Overall, participants achieving PA recommended guidelines show significantly lower odds of experiencing higher current perceived anxiety than those who do not when adjusting for age and gender: (OR, 0.65; 95% CI, 0.55–0.78). Even when the models were adjusted for socioeconomic, health, and COVID-19–related- context variables, the odds for experiencing higher current perceived anxiety remained significantly lower for those following the PA guidelines (OR, 0.66; 95% CI, 0.54–0.79) when compared to those who did not. **Table 2** also shows subgroup analyses, in which women (OR, 0.61; 95% CI, 0.47–0.78), and younger women (OR, 0.59; 95% CI, 0.43–0.79) present the lowest values for the full adjusted model.

**Table 2:** Adjusted odds ratios (95% confidence interval) for higher current perceived anxiety than usual in relation to WHO physical activity guidelines (reference: less than 150 weekly minutes of physical activity) in the entire study population and in age and gender subgroups.

N = 2250	WHO	n	%	Model 1^a^	Model 2^b^	Model 3^c^	Model 4^d^
All	No	1,108	49.2	1	1	1	1
	Yes	1,142	50.8	0.65 (0.55–0.78)	0.66 (0.55–0.78)	0.68 (0.57–0.81)	0.66 (0.54–0.79)
<45 years	No	736	43.8	1	1	1	1
	Yes	944	56.2	0.66 (0.54–0.80)	0.65 (0.54–0.80)	0.69 (0.53–0.81)	0.64 (0.52–0.80)
≥45 years	No	372	65.3	1	1	1	1
	Yes	198	34.7	0.64 (0.44–0.93)	0.63 (0.44–0.92)	0.64 (0.43–0.95)	0.67 (0.45–1.01)
Men	No	465	45.7	1	1	1	1
	Yes	553	54.3	0.67 (0.52–0.87)	0.68 (0.52–0.88)	0.74 (0.52–1.06)	0.75 (0.56–1.01)
Women	No	643	52.2	1	1	1	1
	Yes	589	47.8	0.63 (0.51–0.81)	0.64 (0.51–0.81)	0.62 (0.48–0.79)	0.61 (0.47–0.78)
Men							
<45 years	No	340	41.5	1	1	1	1
	Yes	479	58.5	0.69 (0.52–0.92)	0.70 (0.52–0.93)	0.74 (0.54–1.01)	0.74 (0.54–1.02)
Women <45 years	No	396	45.6	1	1	1	1
	Yes	465	54.0	0.63 (0.48–0.82)	0.63 (0.48–0.82)	0.60 (0.45–0.81)	0.59 (0.43–0.79)
Men							
≥45 years	No	125	62.8	1	1	1	1
	Yes	74	37.2	0.59 (0.32–1.11)	0.60 (0.32–1.13)	0.62 (0.31–1.24)	0.75 (0.36–1.55)
Women							
≥45 years	No	247	66.6	1	1	1	1
	Yes	124	33.4	0.67 (0.42–1.06)	0.65 (0.41–1.03)	0.66 (0.40–1.09)	0.67 (0.41–1.11)

^a^Adjusted for age and gender (all participants), for gender (<45 y, ≥45 y), for age (men, women) and crude model for gender and age subgroups (men < 45 years, women < 45 years, men ≥ 45 years, women ≥ 45 years).

^b^Model 1+ socioeconomic features (civil status, occupation and education).

^c^Model 2+ lifestyle (previous PA, alcohol consumption, smoking habit, sleep time and screen exposure).

^d^Model 3+ COVID-19 isolation context (isolation days, number of partners and proximity to COVID-19).

**Table 3** features borderline significantly lower odds for current perceived worse mood while confined in all the participants who meet the recommended PA guidelines in the final adjusted model (OR, 0.82; 95% CI, 0.68–1.00). Fully adjusted subgroup analyses present significant value for women (OR, 0.74; 95% CI, 0.57-0.95), and borderline significance for younger women (OR, 0.76; 95% CI, 0.56–1.01).

**Table 3:** Adjusted odds ratios (95% confidence interval) for current perceived worse mood in relation to WHO physical activity guidelines (reference: less than 150 weekly minutes of physical activity) in the entire study population and in age and gender subgroups.

N=2250	WHO	n	%	Model 1^a^	Model 2^b^	Model 3^c^	Model 4^d^
All	No	1,108	49.2	1	1	1	1
	Yes	1,142	50.8	0.80 (0.68–0.95)	0.80 (0.67–0.95)	0.84 (0.70–1.00)	0.83 (0.70–1.00)
<45 years	No	736	43.8	1	1	1	1
	Yes	944	56.2	0.83 (0.69–1.01)	0.82 (0.67–1.00)	0.87 (0.71–1.06)	0.86 (0.70–1.05)
≥45 years	No	372	65.3	1	1	1	1
	Yes	198	34.7	0.70 (0.49–1.01)	0.69 (0.48–1.00)	0.73 (0.50–1.06)	0.75 (0.51–1.09)
Men	No	465	45.7	1	1	1	1
	Yes	553	54.3	0.88 (0.69–1.14)	0.89 (0.69–1.16)	0.94 (0.72–1.23)	0.95 (0.73–1.25)
Women	No	643	52.2	1	1	1	1
	Yes	589	47.8	0.74 (0.59–0.93)	0.73 (0.58–0.93)	0.77 (0.61–0.98)	0.77 (0.60–0.97)
Men <45 years	No	340	41.5	1	1	1	1
	Yes	479	58.5	0.93 (0.70–1.23)	0.95 (0.71–1.27)	1.01 (0.75–1.35)	1.01 (0.75–1.36)
Women <45 years	No	396	45.6	1	1	1	1
	Yes	465	54.0	0.75 (0.58–0.99)	0.74 (0.56–0.97)	0.78 (0.59–1.03)	0.76 (0.57–1.01)
Men ≥45 years	No	125	62.8	1	1	1	1
	Yes	74	37.2	0.70 (0.40–1.30)	0.69 (0.37–1.30)	0.69 (0.37–1.30)	0.76 (0.39–1.46)
Women ≥45 years	No	247	66.6	1	1	1	1
	Yes	124	33.4	0.68 (0.44–1.10)	0.70 (0.44–1.10)	0.75 (0.47–1.20)	0.76 (0.47–1.21)

^a^Adjusted for age and gender (all participants), for gender (<45 y, ≥45 y), for age (men, women) and crude model for gender and age subgroups (men < 45 years, women < 45 years, men ≥ 45 years, women ≥ 45 years).

^b^Model 1+ socioeconomic features (civil status, occupation and education).

^c^Model 2+ lifestyle (alcohol consumption, smoking habit, sleep time and screen exposure).

^d^Model 3+ COVID-19 isolation context (isolation days, number of partners and proximity to COVID-19).

## Discussion

The present study in a wide sample of the Spanish adult population found that to achieve a minimum of 150 weekly minutes of PA was significantly associated with lower odds for experiencing higher current perceived anxiety while the COVID-19 confinement. Similarly, participants performing PA guidelines also observed lower odds for current perceived worse mood, although these associations remained solely significant in particular subgroups such as women and women aged below 45 years. These results support the notion that current higher levels of perceived anxiety and worse mood might be mitigated through achieving a minimum amount of weekly PA in this confinement context. In addition, our study provides novel data from a different setting, in which governmental enacted restrictions of free circulation had been implemented for, at least, a period of a month.

### Current Perceived Anxiety

Similarly to our hypothesis, a review of systematic reviews of randomized control trials by Kandola et al. (26), found physical exercise useful to reduce anxiety as well as other mental disorders symptoms. However, these authors pointed at the fact that little is known about how physiological mechanisms of PA influence mental health (26, 27). A meta-analysis by Stubbs et al. (28) observed anxiolytic effects for physical exercise when compared with controls in subjects with diagnosed anxiety or other stress-related disorders.

Regarding the amount of current PA associated with lower current perceived anxiety, previous studies found recommended WHO guidelines to reduce both anxiety symptoms and status in a general population of Irish adults; nevertheless, higher levels of weekly PA showed similar inverse associations with anxiety than the recommended levels (29). Further, PA was observed to significantly reduce anxiety just ten minutes after exercising in both normal and anxiety-diagnosed subjects; although it was a vigorous exercise which more reduced anxiety as regards basal levels (15).

In contrast with the present study, in which women achieving recommended PA guidelines had lower odds for higher current perceived anxiety while confined, Mc Dowell et al. (29) found higher odds for women than for men as regards inverse associations of PA with changes in perceived anxiety; such gender differences might be because both contextual and cultural differences contribute to influencing anxiety (30, 31). Other differences among subgroups could result from the habits of performing PA, since previous studies have indicated a mediator effect of usual exposure to PA over acute anxiety responses (32). Also, the type of PA might play an important role in this association, since recent research has emphasized the effects of specific physical exercise such as high interval intensity training (HIIT) on mental health, showing higher improvements than moderate-intensity continuous training in individuals experiencing mental disorders (33).

### Current Perceived Mood

Our study found a relevant association overall between higher current PA and lower current perceived worse mood among the study sample; however, significant associations were solely observed among specific subgroups. Similarly, a significant body of work has observed improvements in mood state associated with higher levels of PA (34, 35). Furthermore, not only were healthy populations that showed mood enhancement, but also populations with previous conditions (32, 36); even short bouts of PA have been observed to improve mood in older adults (37). Particularly, the acute responses to low-intensity aerobic exercise were those that enhanced mood the most in young women when compared with responses to high-intensity aerobic exercise (37). Interestingly, a recent study found that the largest associations between PA and mental health were for popular team sports, cycling and aerobic and gym activities, as well as durations of 45 min and frequencies of three-five times/week (38). The antidepressant benefits of PA might be explained by the activation of the endocannabinoid system and upregulation of brain-derived neurotrophic factor, as has been observed after 90-minute exercise bouts (39). As indicated by Kandola et al. (27), the effects of PA over depressive symptoms could be also related to social and psychologic mechanisms such as the improvement of self-esteem, sociability, and perceived self-efficacy.

As observed in the present research, differences among subgroups have also been detected in other studies. A close exam to related literature showed previous physical fitness levels, as well as exercise intensity, to influence the acute responses of training over mood responses (40). Comparably reasons may also apply to our study sample. Also, a meta-analysis by Reed et al. (41) focused on acute responses to aerobic exercise over positive activated affect, with higher improvements for those presenting lower levels before the aerobic training; thus, a similar effect might be experienced in participants with worse pre-PA over current perceived mood. In addition, findings from a study by Rocheleau et al. (42) are also noteworthy since they strengthen the notion that gender, exertion level, and training duration might explain differences among study subgroups.

Finally, because COVID-19 settings have been observed to have a greater psychological impact in those with proximal experiences (43), women, students, and people with specific physical symptoms and poor self-rated health status (12), PA could play a critical role in preserving mental health during the confinement period among these subgroups.

### Strengths and Limitations

Strengths of the current study consist of examining a wide and large sample of Spanish adults (i.e. participants representing all the Spanish regions) and using a validated question to estimate PA. Moreover, a wide set of control variables, including novel variables such as the number of days isolated or exposure to COVID-19 have been included in the regression models; although the study did not control other potentially relevant variables (i.e. both physical and pre-diagnosed mental health or either perceived anxiety or mood). In addition, several limitations should be underscored for this study. Firstly, since PA and other variables are self-reported, there is still a chance of an information bias; also, there is a possibility of selection bias (i.e. due to the sampling method we do not know whether the respondents represented the Spanish population in general). Further, of all invited to participate in the survey, 540 individuals (18.9%) declined. In these, no characteristic was observed to be substantially different compared to the group of individuals who agreed to participate (i.e. age, gender, and region). Yet, there is a possibility that those who declined to participate may have had either lower or no benefit from current PA for their current perceived anxiety and mood. Nevertheless, the analyses stratified for age and gender showed important associations between PA adherence to WHO guidelines and lower current perceived anxiety in most of the study subgroups, providing consistency to our findings (i.e. the association between current PA and current perceived anxiety and mood is widely observed across study subgroups). During the present circumstances and due to the necessity to collect data rapidly, it was not possible to use time-consuming methods ensuring a representative sample. Secondly, because young adults are overrepresented in the examined sample, estimated current PA adherence, as well as both current perceived anxiety and mood, might be different than in other older adult population; also, estimated current PA levels might be higher than in the general Spanish population of adults. According to the figures from the National Statistics Institute of Spain (44), the characteristics of this study sample substantially differ from the Spanish general population in variables such as age and education (i.e. the participants of this study are younger and higher educated than the average Spanish general population of adults), hence the findings of the present study should be interpreted in the light of this information. Thirdly, the outcome variables were assessed with a not validated tool, which could lead to an information bias; nevertheless, similar single-item questions have been used in prior research to estimate different mental health conditions, and have shown moderate correlation with validated mental health scales (45, 46). Due to their brevity, single-item questions have been further recommended to apply in specific contexts of illness and frailty, hence, the authors decided to use it in this specific confinement context due to COVID-19 pandemic (47). Last, due to the cross-sectional design, the present study does not allow inferring causal conclusions; thus, randomized controlled trials are further necessary to confirm these findings. However, as the epidemic and subsequent confinement could not be foreseen, it was simply not possible to collect data on pre-confinement conditions to allow for assessment of more causal associations. In closing, because the data of the survey were mainly referred to the first days of the COVID-19 outbreak in Spain, the present results might not be reflecting the worse period of the confinement. (i.e. more days of isolation might lead to higher current perceived anxiety and worse mood). Therefore, future research should focus on how PA adherence might influence perceived anxiety and mood regarding longer-term isolation. Also, the use of larger and validated measurement scales would better contribute to understanding how complex subjects such as anxiety and mood can be affected in a confinement context, and the influence of PA in this relationship.

## Conclusion

The results of the present study indicate that current PA adherence to WHO guidelines in the initial phase of COVID-19 confinement significantly associates with lower current perceived anxiety and lower current perceived worse mood in a large sample of Spanish confined adults. Healthy habits seem to influence both associations which could be stronger among women. Thus, home-based strategies are recommended in this population of adults.

## Data Availability Statement

The raw data supporting the conclusions of this article will be made available by the authors, without undue reservation.

## Ethics Statement

The studies involving human participants were reviewed and approved by Ethics Committee of Research in Humans of the University of Valencia. The patients/participants provided their written informed consent to participate in this study.

## Author Contributions

RL-B, GL-S, JAC, LS, and JC contributed conception and design of the study. RL-B organized the database. RL-B and GL-S performed the statistical analysis; RL-B and GL-S wrote the first draft of the manuscript. JC, LS, LA, YE, and JAC wrote sections of the manuscript. All authors contributed to the article and approved the submitted version.

## Conflict of Interest

The authors declare that the research was conducted in the absence of any commercial or financial relationships that could be construed as a potential conflict of interest.
