# Corrigendum: Association Between Current Physical Activity and Current Perceived Anxiety and Mood in the Initial Phase of COVID-19 Confinement

**DOI:** 10.3389/fpsyt.2021.694760

**Published:** 2021-05-20

**Authors:** Rubén López-Bueno, Joaquín Calatayud, Yasmin Ezzatvar, José A. Casajús, Lee Smith, Lars L. Andersen, Guillermo F. López-Sánchez

**Affiliations:** ^1^Department of Physical Medicine and Nursing, University of Zaragoza, Zaragoza, Spain; ^2^Department of Musculoskeletal Disorders, National Research Centre for the Working Environment, Copenhagen, Denmark; ^3^Exercise Intervention for Health Research Group (EXINH-RG), Department of Physiotherapy, University of Valencia, Valencia, Spain; ^4^Faculty of Health Sciences, University of Zaragoza, Zaragoza, Spain; ^5^Cambridge Centre for Sport and Exercise Science, Anglia Ruskin University, Cambridge, United Kingdom; ^6^Faculty of Sport Sciences, University of Murcia, Murcia, Spain

**Keywords:** physical activity, mental health, Spain, adults, COVID-19

In the original article, the reference for Chen et al. was incorrectly written as Chen P, Mao L, Nassis GP, Harmer P, Ainsworth BE, Li F. Wuhan coronavirus (2019-nCoV): The need to maintain regular physical activity while taking precautions. *J Sport Health Sci* (2020) 9:103–4. doi: 10.1016/j.jshs.2020.02.001. It should be Chen P, Mao L, Nassis GP, Harmer P, Ainsworth BE, Li F. Coronavirus disease (COVID-19): the need to maintain regular physical activity while taking precautions. *J Sport Health Sci*. (2020) 9:103–4. doi: 10.1016/j.jshs.2020.02.001.

The authors apologize for this error and state that this does not change the scientific conclusions of the article in any way. The original article has been updated.

